# Study of Royal Jelly effect on serum levels of IL-4, TNF- α, TGF-B, and IFN-γ in multiple sclerosis patients in comparison with Omega-3; A double-blind, randomized, clinical trial

**DOI:** 10.22038/ajp.2025.26210

**Published:** 2026

**Authors:** Nastaran Majdinasab, Ali Aghaei, Amir Siahpoosh, Rezvan Motamedi, Seyed Ahmad Hosseini, Alireza Malayeri

**Affiliations:** 1 *Department of Neurology, Golestan Hospital, Ahvaz Jundishapur University of Medical Sciences, Ahvaz, Iran*; 2 *Faculty of Pharmacy, Ahvaz Jundishapur University of Medical Sciences, Ahvaz, Iran*; 3 *Medicinal Plant Research Center, Ahvaz Jundishapur University of Medical Sciences, Ahvaz, Iran*; 4 *Nutrition and Metabolic Diseases Research Center, Ahvaz Jundishapur University of Medical Sciences, Ahvaz, Iran*; 5 *Research Unit, Nab’a Al-Hayat Foundation, Najaf, Iraq*

**Keywords:** Multiple sclerosis, Royal Jelly, Omega 3, IFN-γ, TNF- α, TGF-β, IL-4

## Abstract

**Objective:**

Multiple sclerosis (MS) is a debilitating disorder related to damage to the central nervous system (CNS). MS incidence in about 2.3 million people worldwide. Royal jelly (RJ) is a white material with many medicinal properties. Omega-3 (ω−3) is a natural substance that its beneficial effects were demonstrated in several studies performed on MS patients.

**Materials and Methods:**

60 patients referring to the MS Society of Iran were randomly divided into two groups (1:1 ratio) receiving 1-gram RJ and 1-gram ω-3 capsule 1 gr capsules of RJ and 1 gr ω−3 daily in addition to receiving their daily treatment. Then, their information was recorded. Blood samples of all subjects were collected to evaluate the level of IL-4, INF-Y, TGF-B, and TNF-α with Enzyme-linked immunosorbent assay (ELISA) method twice; first, before the intervention and then, received supplements for one month. The duration of treatment was one month, and the patients returned to the center.

**Results:**

The results indicated that RJ, similar to ω−3, could improve the cytokines levels in MS patients. RJ significantly improved TNF-α (p<0.05), and ω−3 significantly decreased TGF-β levels (p<0.05). Both decreased IFN-γ and increased IL-4, but it was more in the group receiving RJ.

**Conclusion:**

According to the findings, it is hoped that using RJ and ω−3 could be helpful in MS patients.

## Introduction

Multiple sclerosis (MS) is a debilitating disorder, which is associated with damage to the central nervous system (CNS). MS incidence is about 2.3 million people around the world, and women are at increased risk by three times (Voskuhl and Momtazee 2017). In this disease, an autoimmune reaction destroys the brain's nerve along the spinal cord (Zarei et al. 2019). CD8+ T-cells and B-cells are the main cells that infiltrate to the blood-brain barrier (BBB) and cause the formation of focal white matter lesions both in the spinal cord and brain (Lassiter et al. 2020). The primary cause of this disease is the inflammatory response, which contributes to myelin damage and cell death (Chen et al. 2020). Earlier study has revealed that MS patients have increased concentrations of some proinflammatory Factors such as IL-1, IL-12, IL-17, IL-22, and TNF-α. They have also decreased concentrations of anti-inflammatory parameters such as IL-4 and IL-10 (Zarrabi et al. 2021). Further, *in vitro* investigations have informed that T lymphocytes can destroy myelin and glial cells (Hirose et al. 2020). The bindings cause generation of IL-1, IL-6, IL-12, IL-18, IL-23, accelerating the stimulation and development of Th1 plus Th17 cells (Govindarajan et al. 2020). In other words, patients with MS have greater levels of IL-22 and IFN-*γ* (Waite and Skokos 2012). 

Royal jelly (RJ) which is the main source of food for queen bees, is secreted by the hypopharyngeal and mandibular glands (Bălan et al. 2020). There is evidence demonstrating that RJ has antioxidant, anti-inflammatory, neurotrophic, hypotensive, antidiabetic, and antirheumatic characteristics. (Shirakawa et al. 2020);(Ali and Kunugi 2020). The chemical composition of royal jelly is mainly water, followed by protein, carbohydrates, lipids, vitamins, and minerals. The most frequent fatty acids available in the RJ contain 10-hydroxy-2-decenoic acid (10H2DA) and 10-hydroxydecanoic acid (10-HDA). 10-HDA is a potential inhibitory factor of matrix metalloproteinases (MMPs) in many inflammatory disorders. RJ reduces inflammatory factors including IL-12, TNF-α, IL-6, in addition IL-1 ((Erem et al. 2006);(Kohno et al. 2004);(Takahashi et al. 2012). On the other hand, RJ has a positive effect on autoimmune and anti-inflammatory disease (Kohno et al. 2004). 

Polyunsaturated fatty acids (PUFAs) contain two main groups: ω-3 PUFAs including eicosapentaenoic acid (20:5; ω-3; EPA), α-linolenic acid (18:3; ω-3; α-LA), and docosahexaenoic acid (22:6; ω-3; DHA); and ω-6 PUFAs (Levant 2013), with fish and seafood containing great levels of ω-3 (Jelinek et al. 2013). Studies have shown that ω-3 effectively prevents inflammation of neurons by regulating neurotransmitters of dopamine and serotonin as well as hippocampus brain-derived neurotrophic factor (BDNF) (Levant 2013); (Jelinek et al. 2013). In the studies on ω-3 effects on patients with MS, its beneficial effects were proven. For example, a cohort of MS patients from various countries indicated that ω-3 enhanced quality of life, while reducing activity of the disease and disability (Jelinek et al. 2013). Finally, ω-3 has been reported as a potential factor to reduce the concentration of both cytokines and nitric oxide (NO) in MS patients (Riccio et al. 2016).

In this study, we tried to evaluate the probable impact of RJ plus ω−3 fatty acid on serum cytokines in MS patients. Considering the proven and beneficial effects of RJ, the effect of this supplement was considered in the treatment of MS, and ω-3 was used for comparison, whose positive effect on MS has been proven in earlier reports.

## Materials and Methods

### Participants

This research was a randomized, double-blind, controlled trial. 60 patients were enrolled from Ahvaz Jundishapur University of Medical Science neurology department, Iran. This study was approved by the Iranian Registry of Clinical Trials (Protocol number: IRCT20181026041466N4). Informed consent was taken from every subject before the study, corresponding to the ethical code of the organization. Recognition numbers were given to confirm subject confidentiality.

The inclusion criteria: 1) diagnosis of relapsing-remitting MS (RRMS) based on neurological examinations and magnetic resonance imaging (MRI) by a neurologist, 2) age at 15-55 years, 3) Expanded Disability Status Scale (EDSS) score less than 6, 4) no other neurological diseases, such as stroke or psychiatry disorder, such as depression, 5) no pregnancy for women, 6) no diseases such as malignancy-autoimmune diseases including lupus or rheumatoid arthritis and heart disease, 7) absence of any new attack in the last month and use of any corticosteroid medication.

The exclusion criteria included: 1) occurrence of supplement side effects (such as nausea, stomachache, and diarrhea); 2) dissatisfaction with participating in the study (the patient's dissatisfaction with the supplement such as bad taste, allergenic, and sensation); 3) No drug use for one week; 4) pregnancy.

### Randomization and blinding

Subjects were allotted at random in a 1:1 ratio to receive ω−3 or RJ, based on a computer-generated randomization sequence (blocks of 2–4). Capsules were matching in form, packaging, and labeling to guarantee hiding between the ω−3 and the RJ. Physicians and patients were blind to the medications.

### Clinical trial

In this study, 1 g capsules of RJ (made by Marines, Spain) and 1 g ω−3 (made by Alfa-American company) were used. The patients referring to Iran MS Center were randomly divided into two groups. Then, their information was recorded. 

For randomization, the drug boxes previously encoded without the researcher's awareness were randomly delivered to the patients. The patients received the encoded drug packages (1 g of RJ or ω−3) daily in addition to receiving their daily treatment (250 *μ*g interferon beta-1b SC every other day). The duration of treatment was one month, and the patients returned to the center. Peripheral blood samples of all subjects were collected two times; before the intervention, 6 ml of fasting blood samples were collected in EDTA tubes, and at the end of the study in the similar condition.

Standard Ficoll (lymphosep) 1.077 gr/ml centrifugation (25 min, 450 × g) process was used to isolate peripheral blood mononuclear cells (PBMCs). The mean number of 1.18 × 10^6^ ± 0.12 PBMCs was isolated per ml of whole blood. Specifically, 5×10^6^ isolated PBMCs were cultured in 5 ml Roswell Park Memorial Institute (RPMI) 1640 medium containing 10% heat-inactivated fetal calf serum (FCS), and subsequently, 200 mM of glutamine and 100 U/ml of penicillin were added to the medium. It was followed by addition of Ten ng/ml of phorbol myristate acetate (PMA) and incubation 48 hours at 37° centigrade for 48 and 5 percent CO_2_.

### Measurements of outcomes

#### Cytokine evaluation

For calculate the quantities of IL-4, INF-Y, TGF-B, TNF-α in the supernatant of PBMCs culture and serum, ELISA method was used. 100 μl of the initial antibodies were coated in wells of a 96-well plate overnight. Subsequently, the wells were washed with PBS containing 0.005% Tween to eliminate extra antibodies and incubated with blocking antibodies for one hour on a shaker. Next, 100 μl of standards or samples were inserted into the wells and incubated on a shaker for one hour. Then, the wells were first incubated with 100 μl of biotinylated antibody for one hour. After that, 100 microliters of Avidin-Biotin-Peroxidase (ABC) complex was combined and incubated for thirty minutes. Then, 100 μl of tetra methyl benzidine (TMB) substrate solution was added to the wells. Finally, twenty-five minutes later, for determining the absorbance the Medgenix ELISA reader was used at 450 nm. For evaluating the concentration, the standard calibration lines were used for of the samples using the Softmax software.

### Safety

The adverse effects (AE) of ω−3 and RJ were documented during the study. A severe AE was described as any consequence that would cause fatality or require hospitalization.

### Statistical analysis

The results of the required tests, questionnaires, and data were collected and used SPSS. The normal distribution of data was approved using the Smirnov-Kolmogorov test. Descriptive statistics were used to provide mean and standard deviation, while independent and paired t-tests were employed to compare the mean of the two groups before and after the intervention in the case of the normal variables. Wilcoxon and Mann-Whitney tests were used in case of variability. The significance level of 0.05 and the SPSS 22 were utilized.

## Results

**Figure 1:**
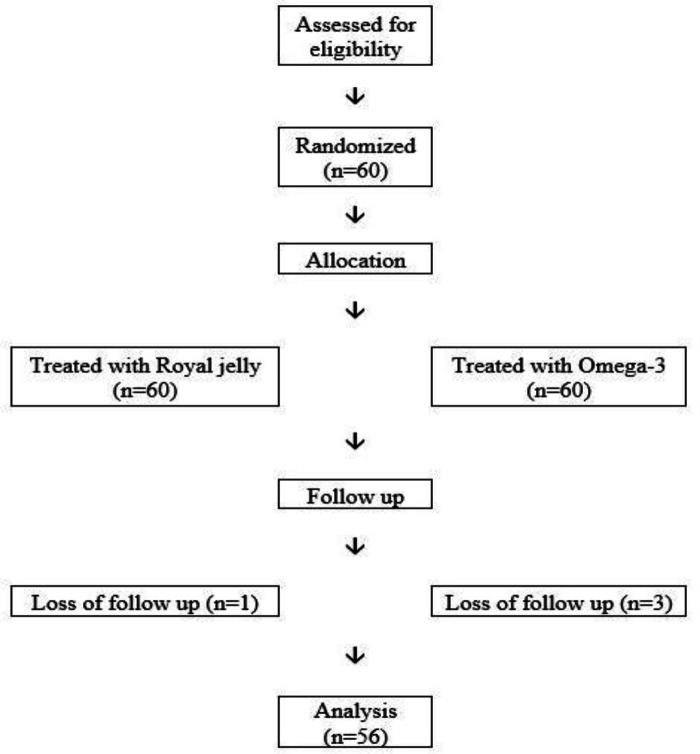
Flowchart of the study

In this study, 60 RRMS patients, aged 15-55 years, were studied, with 45 being female and 15 being male (Table 1). One patient was excluded from the study due to an allergy to the supplement, and three others were excluded due to personal issues; ultimately, 56 people finished the study (Figure 1). 

**Table 1:** The demographic information of the patients

Omega-3 (patient)	Royal Jelly (Patient)		
6	9	Man	Gender
24	21	Woman	
14	10	Single	Martial State
15	18	Married	
1	2	Divorced	
1	0	< 20 years	Age
24	21	20-40 years	
6	9	> 40 years	

Regarding the serum levels of inflammatory cytokines, in the RJ group, serum TNF-α levels dropped by 9 pg/ml from baseline (p<0.05), while in the ω−3 group, there was a reduction of 4.7 pg/ml. However, serum IFN-γ levels post-intervention decreased by 0.76 pg/ml in the RJ group when compared to baseline levels and fell by 0.24 pg/ml in the ω−3 group. In contrast, the serum TGF-β level of the RJ group after the intervention increased by 1.91 ng/ml compared to baseline levels and dropped by 15.15 ng/ml in the ω-3 group (p<0.05). IL-4 was elevated in both groups (0.2 pg/ml in the RJ group and 0.24 pg/ml in the ω−3 group) compared to the control.

**Figure 2:**
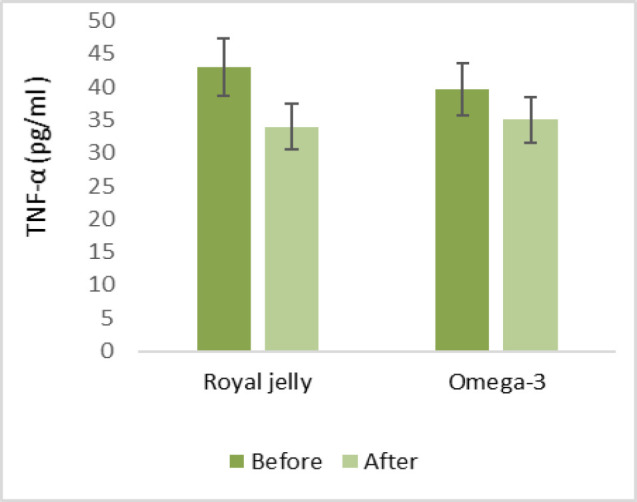
The effect of Royal jelly and Omega-3 on serum levels of TNF-α. (*showed statistical difference between before and after treatment (*p<0.05)

**Figure 3:**
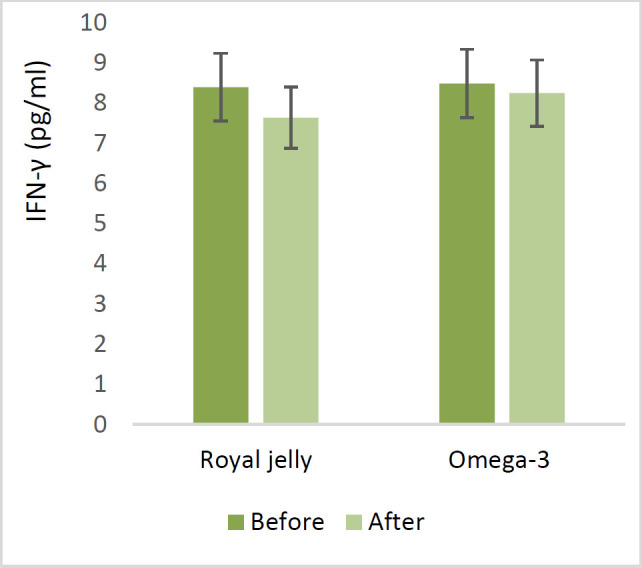
The effect of Royal jelly and Omega-3 on serum levels of IFN-γ. (There is not significantly difference between the beginning and end of the study or between two groups.)

**Figure 4:**
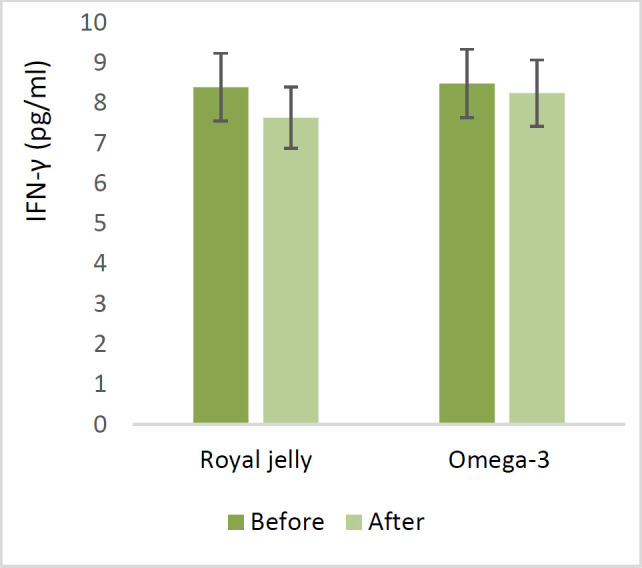
The effect of Royal jelly and Omega-3 on serum levels of IL-4. (There is not significantly difference between the beginning and end of the study or between two groups

**Figure 5:**
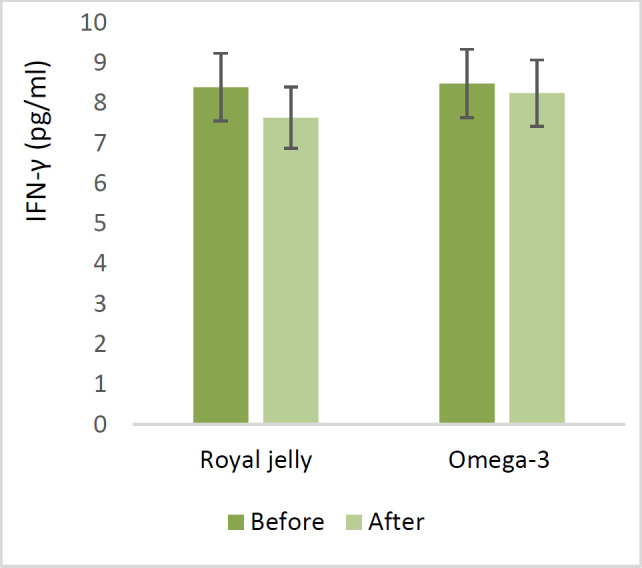
The effect of Royal jelly and Omega-3 on serum levels of TGF-β. (*showed statistical difference between before and after treatment (*p<0.05)

Regarding the serum levels of inflammatory cytokines, in the RJ group, serum TNF-α levels dropped by 9 pg/ml from baseline (p<0.05), while in the ω−3 group, there was a reduction of 4.7 pg/ml (Figure 2). However, serum IFN-γ levels post-intervention decreased by 0.76 pg/ml in the RJ group when compared to baseline levels and fell by 0.24 pg/ml in the ω−3 group (Figure 3). In contrast, IL-4 was elevated in both groups (0.2 pg/ml in the RJ group and 0.24 pg/ml in the ω−3 group) compared to the control. (Figure 4). The serum TGF-β level of the RJ group after the intervention increased by 1.91 ng/ml compared to baseline levels and dropped by 15.15 ng/ml in the ω-3 group (p<0.05) (Figure 5). 

## Discussion

MS is a chronic autoimmune disorder that causes damage to the nervous system. 

In this disease, an autoimmune reaction destroys the brain's nerve along the spinal cord (Zarei et al. 2019). The major cause of this disease is the inflammatory response, which contributes to myelin damage and cell death (Chen et al. 2020). Previous research has shown that in MS patients, the concentration of some proinflammatory Factors Like IL-1, IL-12, and TNF-α is elevated. Also, they have lowered concentrations of anti-inflammatory Factors like IL-4 and IL-10 (Zarrabi et al. 2021). Treatment of MS is centered on reducing the recurrence of disease symptoms, mostly in RRMS or SPMS patients (Zarei et al. 2019). The high number of novel treatments promises to cease the development of new lesions (Bove and Green 2017). 

The effect of RJ has been proven to enhance learning capacity, improve memory, and sustainability against fatigue in various studies (Kamakura et al. 2001; Morris and Stare 1993). It also facilitates neurogenesis and increases oxygen delivery to the brain tissue (Hattori et al. 2007). RJ contains phosphorus compounds, especially acetylcholine, one of the carriers of neuronal messages from one cell to the next and can be effective in neurological diseases (Barker et al. 1959). RJ reduces inflammatory Factors such as IL-12, TNF-α, IL-6 and IL-1, inhibits the production of NO products, declines the stimulation of NF-kB, and inhibits generation of cytokines in response to IFN- γ activity (Erem et al. 2006); (Kohno et al. 2004) ; (Takahashi et al. 2012). The results of this study confirmed previous similar studies that RJ can reduce TNF-α and IFN-γ. For example, Moulai et al. showed that royal jelly combined with exercise significantly reduced the TNF-α factor in patients with MS (Molaei et al. 2019). Miyata et al. also showed that RJ supplementation reduced the serum levels of TNF-α and IFN-γ significantly in patients (Ahmad et al. 2020; Miyata et al. 2020). Fish oil and ω-3 PUFA inhibit the production of proinflammatory transcription factor nuclear factor-kB (NF-kB). Due to its anti-inflammatory and neuroprotective properties, administering fish oil was associated with lowered concentration of cytokines and NO in RRMS patients who receive IFN-(Riccio et al. 2016); (Simonetto et al. 2019). In this study, ω−3 reduced TNF-α and IFN-γ, while various results have been obtained in similar studies, which can be caused by the difference in the type and amount of omega-3 supplements consumed, the number and type of patients with MS, etc. For example, Ramirez et al. showed that ω-3 has a significant effect on reducing TNF-α levels. It also stimulates IFN-γ serum levels (Ramirez-Ramirez et al. 2013). These results are in accordance with Sadaghat et al., who stated that a diet low in omega-3 may increase the risk of MS (Sedaghat et al. 2016). Contrary to them, Zandi-Esfahan et al. reported that taking 1 gram of fish oil did not reduce TNF-α, IL1β, IL6, and IFN-γ serum levels compared to placebo (Zandi-Esfahan et al. 2017). Similar results were reported by Wergeland et al., who found no evidence of PUFA benefit in MS patients (Wergeland et al. 2012).

Finally, this study indicated that royal jelly, similar to ω−3, could improve the cytokine levels in MS patients without any adverse effects. Both reduced TNF-α, IFN-γ and increased IL-4 levels. However, it was more improvement in the group receiving RJ. RJ can significantly improve TNF-α, and omega-3 can significantly improve TGF-β levels. 
